# The effect of social deprivation on the dynamic of the SARS-CoV-2 infection in France

**DOI:** 10.1093/eurpub/ckac131.111

**Published:** 2022-10-25

**Authors:** S Smaili, C Pelat, E Chatignoux, M Kelly-Irving, C Delpierre, S Vandentorren

**Affiliations:** 1 Data Science Division, Sante Publique France, Saint Maurice, France; 2 IFERISS, University of Toulouse Jean Jaurès, Toulouse, France; 3 CERPOP Inserm UMR 1295, University of Toulouse Paul Sabatier, Toulouse, France; 4 Inserm UMR 1219 Bordeaux Population Health, University of Bordeaux, Bordeaux, France

## Abstract

**Background:**

The association between health inequalities and the SARS-CoV-2 infection dynamic remains to be studied in France. The objective of this study was to analyse the relationship between an area-based deprivation indicator and SARS-CoV-2 infection indicators, during four epidemic waves running from August 4th 2020 to January 27th 2021 (second wave), January 28th to June 24th 2021 (third wave), June 25th to October 28th 2021 (fourth wave), and October 29th 2021 to March 29th 2022 (fifth wave).

**Methods:**

We analysed weekly indicators of SARS-CoV-2 infection, extracted from the national testing information system: incidence, positivity and testing rates. The associations of these outcomes with the European Deprivation Index (EDI) quintiles were estimated with negative binomial generalized additive models adjusted for epidemic waves, population density (sparsely, moderately, densely populated), region (random effect) and interactions between epidemic waves and the variables EDI, population density, and region.

**Results:**

The most deprived areas had a higher positivity rate than the least deprived ones during the second, third and fourth waves, but a lower rate during the fifth wave. They also had higher incidence during the third and fourth waves, but a similar incidence than the least deprived areas during the second wave, and even a lower rate during the fifth wave. The testing rate was lower in the most deprived areas than elsewhere, irrespective of the epidemic waves.

**Conclusions:**

People living in the most deprived areas were less likely to be tested and more likely to test positive than people living in less deprived areas. The lower incidence, positivity and testing rates during the fifth wave in the most deprived areas may be explained by the enacted change in policy whereby screening tests were no longer free. These findings may reflect structural differences in access to care and lower capacity to benefit from prevention measures by deprived populations.

**Key messages:**

• People living in the most deprived areas were less likely to be tested for COVID-19 irrespective of the epidemic waves.

• Health authorities should address the issues of social inequalities more rapidly and target prevention strategy to disadvantaged populations.

